# Early-onset autoimmunity associated with *SOCS1* haploinsufficiency

**DOI:** 10.1038/s41467-020-18925-4

**Published:** 2020-10-21

**Authors:** Jérôme Hadjadj, Carla Noemi Castro, Maud Tusseau, Marie-Claude Stolzenberg, Fabienne Mazerolles, Nathalie Aladjidi, Martin Armstrong, Houman Ashrafian, Ioana Cutcutache, Georg Ebetsberger-Dachs, Katherine S. Elliott, Isabelle Durieu, Nicole Fabien, Mathieu Fusaro, Maximilian Heeg, Yohan Schmitt, Marc Bras, Julian C. Knight, Jean-Christophe Lega, Gaetan Lesca, Anne-Laure Mathieu, Marion Moreews, Baptiste Moreira, Audrey Nosbaum, Matthew Page, Cécile Picard, T. Ronan Leahy, Isabelle Rouvet, Ethel Ryan, Damien Sanlaville, Klaus Schwarz, Andrew Skelton, Jean-Francois Viallard, Sebastien Viel, Marine Villard, Isabelle Callebaut, Capucine Picard, Thierry Walzer, Stephan Ehl, Alain Fischer, Bénédicte Neven, Alexandre Belot, Frédéric Rieux-Laucat

**Affiliations:** 1 Université de Paris, Imagine institute, laboratory of Immunogenetics of Pediatric Autoimmune Diseases, INSERM UMR 1163, 24 boulevard du Montparnasse, 75015 Paris, France; 2 Université de Paris, IHU-Imagine, 24 boulevard du Montparnasse, Paris, 75015 France; 3 Institute for Immunodeficiency, Center for Chronic Immunodeficiency, Medical Center - University of Freiburg, Faculty of Medicine, University of Freiburg, Freiburg, Germany; 4 Centre International de Recherche en Infectiologie, CIRI, Inserm, U1111, Université Claude Bernard Lyon 1, CNRS, UMR5308, École Normale Supérieure de Lyon, University of Lyon, Lyon, France; 5 Centre de Référence National des Cytopénies Auto-immunes de l’Enfant (CEREVANCE), CIC 1401, Inserm CICP, Bordeaux, France; 6 Pediatric Oncology Hematology Unit, University Hospital, place Amélie Raba Léon, CIC 1401, Inserm, CICP, Bordeaux, France; 7 Translational Medicine, UCB Pharma, Braine-l’Alleud, Belgium; 8 Experimental Therapeutics, Radcliffe Department of Medicine, University of Oxford, Oxford, United Kingdom; 9 Translational Medicine, UCB Pharma, Slough, United Kingdom; 10 Department of Pediatrics, Kepler University Hospital and School of Medicine, Johannes Kepler University, Linz, Austria; 11 Wellcome Centre for Human Genetics, University of Oxford, Oxford, UK; 12 Internal Medicine and Vascular Pathology Department, Adult Cystic Fibrosis Center, Groupement Hospitalier Lyon-Sud, Hospices Civils de Lyon, Pierre-Bénite, France; 13 EA 7425 HESPER. Université de Lyon, Lyon, France; 14 Immunology laboratory; Centre Hospitalier Lyon Sud, Hospices Civils de Lyon, Lyon, France; 15 Study Center for Primary Immunodeficiencies, AP-HP, Necker Hospital for Sick Children, Paris, France; 16 Genomic Core Facility, INSERM UMR1163, Imagine Institute, Paris, France; 17 Department of Internal and Vascular Medicine, Centre Hospitalier Lyon Sud, Hospices Civils de Lyon, Lyon, France; 18 National Referee Centre for Pediatric-Onset Rheumatism and Autoimmune Diseases (RAISE), Lyon, France; 19 UMR 5558, Equipe Evaluation et Modélisation des Effets Thérapeutiques, Laboratoire de Biométrie et Biologie Evolutive, CNRS, Claude Bernard University Lyon 1, Lyon, France; 20 Service de Génétique, Hospices Civils de Lyon - GHE, and Institut Neuromyogène, CNRS UMR 5310 - INSERM U1217, Université de Lyon, Université Claude Bernard Lyon 1, Lyon, France; 21 Immunology Laboratory, Necker Children’s Hospital, Assistance Publique-Hôpitaux de Paris, Paris, France; 22 Allergy and Clinical Immunology department, Groupement Hospitalier Lyon-Sud, Hospices Civils de Lyon, Pierre-Bénite, France; 23 Institut de Pathologie Multisite, Groupement Hospitalier Est, Hospices Civils de Lyon, UCBL Lyon 1 University, Lyon, France; 24 Department of Paediatric Immunology and Infectious Diseases, Children’s Health Ireland at Crumlin, Dublin, Ireland; 25 Centre de biotechnologie cellulaire et Biothèque, Groupe Hospitalier Est, Hospices Civils de Lyon, 69677 Bron, France; 26 Department of Paediatrics, University Hospital Galway, Co, Galway, Ireland; 27 Institute for Transfusion Medicin, University Ulm and Institute for Clinical Transfusion Medicine and Immunogenetics Ulm, German Red Cross Blood Service Baden-Württemberg-Hessen, 89081 Ulm, Germany; 28 Département de Médecine Interne et Maladies Infectieuses, Centre Hospitalier Universitaire Haut Lévêque, Université de Bordeaux, Pessac, France; 29 Service d’Immunologie Biologique, Groupement Hospitalier Lyon-Sud, Hospices Civils de Lyon, Pierre-Bénite, France; 30 Sorbonne Université, Muséum National d’Histoire Naturelle, Centre National de la Recherche Scientifique Unité Mixte de Recherche 7590, Institut de Minéralogie, de Physique des Matériaux et de Cosmochimie, Paris, France; 31 Université de Paris, Imagine institute, laboratory of Iymphocyte activation and susceptibility to EBV, INSERM UMR 1163, 24 boulevard du Montparnasse, Paris, 75015 France; 32 CIBSS - Centre for Integrative Biological Signalling Studies, University of Freiburg, Freiburg, Germany; 33 Paediatric Immuno-Haematology and Rheumatology Department, Necker-Enfants Malades University Hospital, Assistance Publique-Hôpitaux de Paris, 75015 Paris, France; 34 Collège de France, Paris, France; 35 Hospices Civils de Lyon, Paediatric Nephrology, Rheumatology, Dermatology Unit, Mother and Children University Hospital, Bron, France

**Keywords:** Disease genetics, Autoimmunity, Interferons

## Abstract

Autoimmunity can occur when a checkpoint of self-tolerance fails. The study of familial autoimmune diseases can reveal pathophysiological mechanisms involved in more common autoimmune diseases. Here, by whole-exome/genome sequencing we identify heterozygous, autosomal-dominant, germline loss-of-function mutations in the *SOCS1* gene in ten patients from five unrelated families with early onset autoimmune manifestations. The intracellular protein SOCS1 is known to downregulate cytokine signaling by inhibiting the JAK-STAT pathway. Accordingly, patient-derived lymphocytes exhibit increased STAT activation in vitro in response to interferon-γ, IL-2 and IL-4 that is reverted by the JAK1/JAK2 inhibitor ruxolitinib. This effect is associated with a series of in vitro and in vivo immune abnormalities consistent with lymphocyte hyperactivity. Hence, *SOCS1* haploinsufficiency causes a dominantly inherited predisposition to early onset autoimmune diseases related to cytokine hypersensitivity of immune cells.

## Abstract (web summary)

SOCS1 is a potent suppressor of JAK-STAT signalling responses to IFNγ and γ-chain cytokines and thereby limits inflammation. Here the authors identify and characterize heterozygous SOCS1 mutations in 10 patients from 5 unrelated families with autoimmune diseases.

## Introduction

The immune system is tightly regulated by many central and peripheral checkpoints, in order to prevent reactivity to self-antigens. Their defects can lead to the expansion of self-reactive B and T lymphocytes, immune system overactivation, and thus autoimmune diseases^1^. Several monogenic defects leading to the breakdown of immune tolerance and autoimmunity have been identified, showing that a number of proteins are involved in non-redundant inhibitory checkpoints^2^. Moreover, the study of familial autoimmune diseases can reveal pathophysiological mechanisms also involved in more common autoimmune diseases^2^.

Cytokines, including interleukins and interferons (IFNs), participate in the development, differentiation, and effector functions of lymphoid and myeloid cells^3^. To do so, cytokines activate the Janus kinase (JAK)-STAT pathway that drives the transcription of cytokine-inducible genes^3^. Prolonged signaling (particularly by proinflammatory cytokines) can be harmful, requiring tight regulation of these pathways. The intracellular, inducible suppressors of cytokine signaling (SOCS) family of proteins is part of this negative-feedback system^4^. The SOCS family has eight members, all of which have a conserved central Src-homology 2 (SH2) domain (binding to JAKs’ phosphotyrosine-containing sequences) and a short C-terminal SOCS box (causing the ubiquitination and proteasomal degradation of captured substrates)^4^. SOCS1 is the most potent member of the SOCS family, and is the primary downregulator of cytokines involved in immune responses—particularly those that signal through the γc-chain (the IL-2 family) and interferons^4,5^. In addition to exerting ubiquitin ligase activity, SOCS1 directly inhibits JAK kinase activity through its kinase inhibitory region (KIR domain)^5^.

Here, using whole-exome/genome sequencing, we identify heterozygous germline *SOCS1* mutations in ten patients from five unrelated families with early onset autoimmune manifestations and lymphoproliferation resulting in impaired SOCS1 function, increased STAT activation, and cytokine hypersensitivity of immune cells.

## Results

### Clinical features and immunological findings

**Fig. 1:**
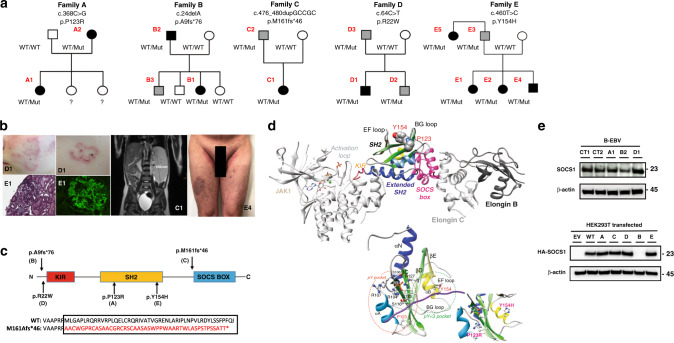
Pedigrees and genetics of families with *SOCS1* mutations. **a** Pedigrees of families with *SOCS1* mutations. Squares: males; circles: females; black: affected mutation carriers; gray: unaffected mutation carriers. WT: wild-type *SOCS1* allele. **b** Clinical manifestations in patients with *SOCS1* mutations. Discoid lupus erythematosus (D1, upper left), active lupus nephritis (E1, lower left) with segmental cellular crescent associated with mesangial hypercellularity (Masson’s trichrome × 400) and glomerular capillary wall and mesangial C1q deposition by immunofluorescence microscopy, abdominal MRI showing splenomegaly (C1, middle), and plaque, intertrigous and guttata psoriasis (E4, right). **c** SOCS1 protein domains and locations of the mutations (upper panel, black arrows). The kinase inhibitory region (KIR) functions as a pseudosubstrate that can inhibit the tyrosine kinase activity of Janus kinase (JAK) proteins. The SRC-homology 2 (SH2) domain binds the activation loop of the JAK proteins’ catalytic domain. The SOCS box recruits the ubiquitin-transferase system and initiates the proteasomal degradation of JAK proteins. The SOCS1 M161Afs*46 mutant leads to a predicted 46-residue neopeptide in the SOCS box domain three amino acids shorter than the wild-type protein (lower panel). **d** Top panel: position of the P123 and Y154 in human SOCS1 within a 3D model of the JAK1/SOCS1 complex. Bottom panel: a model of the human SOCS1’s SH2 domain. The two mutated amino acids (P123R and Y154H) are highlighted in the phosphotyrosine peptide binding groove (the pY and pY+3 pockets). The possible location of a phosphotyrosine peptide is shown in purple. **e** SOCS1 protein expression in patient-derived cells and transfected cells. Top**:** Western blot (WB) analysis of lysates from Epstein-Barr-virus (EBV)-tranformed B cells from patients A1, B2, and D1 and from two healthy controls (CT1 and CT2), following incubation with anti-SOCS1 antibodies (upper panel) or anti-actin antibodies as a loading control (lower panel). Bottom: HEK293T cells transiently transfected with an empty vector (EV), a vector coding for hemagglutinin (HA)-tagged wild-type (WT) SOCS1 protein, or vectors coding for the five HA-tagged mutant SOCS1 proteins. Lysates were incubated with anti-HA antibodies (upper panel) or anti-actin antibodies as a loading control (lower panel). Data are representative of two independent experiments.

We assessed ten patients from five unrelated kindreds presenting early onset autoimmune manifestations and *SOCS1* mutations (see below) (Fig. 1a). The patients’ main characteristics are summarized in Table 1 and cases are described in more detail in the Supplementary Notes. With the exception of one (E5), the first symptoms appeared during childhood (median (range) age: 7.5 years (2–44)) in all patients. Five patients (A1, A2, B1, C1, and E2) had autoimmune cytopenia, four patients (A2, B2, E4, and E5) had organ-specific autoimmunity (thyroiditis, coeliac disease, psoriasis, spondyloarthritis, and hepatitis), and two patients (D1 and E1) met the diagnostic criteria for systemic lupus erythematosus (SLE) with skin (discoid lupus) and kidney (lupus glomerulonephritis) involvement (Fig. 1b). Two patients (B1, and C1) had benign lymphoproliferation (lymphadenopathy and/or hepatosplenomegaly (Fig. 1b)), whereas one (B2) developed Hodgkin lymphoma. None had infectious complications, with the exception of recurrent bronchopulmonary infections in B1.

**Table 1:** Clinical phenotype of patients with *SOCS1* mutations.

Patient	Gender	Genotype	Age at onset (years)	Autoimmune manifestations	Lymphoproliferation	Infections	Therapy
*Family A*							
A1	F	WT/P123R	2	Severe ITP	No	No	Corticosteroids, IVIg, TPOr agonist, MMF
A2	F	WT/P123R	6	ITP Thyroiditis Polyarthritis	No	No	Corticosteroids Hormonal substitution
*Family B*							
B1	F	WT/A9Pfs*76	5	Evans syndrome	Adenopathy Splenomegaly	Bronchopulmonary	Corticosteroids, IVIg, Rapamycine
B2	M	WT/A9Pfs*76	3	Coeliac disease Psoriasis	Hodgkin lymphoma	No	Topical treatment
*Familiy C*							
C1	F	WT/M161Afs*46	3	Evans syndrome	Adenopathy Hepatomegaly Splenomegaly	No	Corticosteroids, MMF
*Family D*							
D1	M	WT/R22W	16	Systemic lupus: glomerulonephritis	No	No	Corticosteroids, Hydroxychloroquine, MMF
*Family E*							
E1	F	WT/Y154H	9	Systemic lupus: polyarthritis, glomerulonephritis	No	No	Corticosteroids, Hydroxychloroquine, Methotrexate, Cyclophosphamide, MMF, Baricitinib
E2	F	WT/Y154H	16	ITP	No	No	Corticosteroids, IVIg, Hydroxychloroquine, Azathioprin, Rituximab, Splenectomy
E4	M	WT/Y154H	15	Psoriasis	No	No	Topical treatment
E5	F	WT/Y154H	44	Psoriasis, Spondyloarthritis, Autoimmune hepatitis and pancreatitis	No	No	Corticosteroids, Hydroxychloroquine, Methotrexate, Anti-TNFα therapy

*F* female, *M* male, *WT* wild-type, *ITP* immune thrombocytopenia, *IVIg* intravenous immunoglobulin, *TPOr* TPOr receptor, *MMF* mycophenolate mofetil, *TNF* tumor necrosis factor.

Immunological investigations were performed on peripheral blood mononuclear cell samples from all patients (Supplementary Table 1). The marginal zone and/or switched memory B cell counts were both low in seven patients (Supplementary Fig. 1A and Supplementary Table 1), whereas IgG levels were normal. Elevated CD21^low^ CD38^low^B-cell counts (that may include autoreactive B cells) were observed in seven out of eight patients (Supplementary Fig. 1B). Eight of the nine patients tested had autoantibodies, including antinuclear autoantibodies (*n* = 5), anti-DNA antibodies (*n* = 3), antinuclear antigen antibodies (anti-SSA, anti-RNP, anti-SCL, *n* = 5), and a positive Coombs test (*n* = 3). It is noteworthy that plasma B-cell activating factor (BAFF) levels were elevated in five of the eight patients tested (Supplementary Table 1). Although the T and NK cell counts were variable, no cell subsets specific abnormalities were found.

### Identification of *SOCS1* mutations

Whole-exome or genome sequencing was performed on whole blood DNA samples from six patients (A1, A2, B1, C1, D1, and E1). Heterozygous germline *SOCS1* mutations were identified in all six patients, including three missense mutations in families A (c.368 C > G, p.P123R), D (c.64 C > T, p.R22W) and E (c.460 T > C, p.Y154H), respectively, and two frameshift mutations in families B (c.24delA, p.A9fs*76) and C (c.476_480dupGCCGC, pM161fs*46) (Fig. 1a, Supplementary Fig. 2A). All *SOCS1* variants were predicted to be deleterious, with a high CADD (Combined Annotation-Dependent Depletion) score above the cutoff score of 11.63 indicating mutational significance^6^, and were absent or very rare in public gene mutation databases (Supplementary Fig. 2b). Two mutations (in families B and D) were located at the 5′ end of the kinase inhibitory region, two (in families A and E) were located in the SH2 domain, and one (in family C) was located at the 5′ end of the SOCS-box domain (Fig. 1c). Three distinct missense variants (in families A, D, and E) affected phylogenetically conserved amino acids (Supplementary Fig. 2C). It is noteworthy that residues P123 and Y154 were previously reported as a site of somatic mutations in B cell lymphomas^7,8^. An analysis of the SOCS1 three-dimensional (3D) protein structure showed that P123 and Y154 are located within the groove (Fig. 1d) that binds phosphorylated JAK peptides with high affinity^5^. 3D structure information was not available for R22, belonging to a disordered segment. In view of its position (upstream of the kinase inhibitory region), this mutation could perhaps perturb binding to JAK peptides. Details of the SOCS1 mutants’ 3D modeling and analysis are given in the Supplementary Notes and in Supplementary Fig. 3-4. Mutations were then identified in four additional patients (B2, E2, E4, and E5) as well as five apparently healthy carriers. The familial segregation thus indicated autosomal-dominant inheritance with incomplete clinical penetrance (Fig. 1a).

The impact of *SOCS1* mutations on protein expression was analyzed using patient-derived EBV-transformed B cell lines and HEK293T cells transfected with the various *SOCS1* alleles. Ectopic expression of the p.A9Pfs*76 mutation (family B) did not yield detectable protein levels (Fig. 1e bottom and Supplementary Fig. 5) while lower expression was detected in the corresponding EBV-B mutant cell line, indicating haploinsufficiency (Fig. 1e top). The frameshift mutation in family C yielded a detectable protein containing a predicted 46-residues neopeptide within the SOCS box domain and a predicted premature stop codon removing the last three amino acid residues (Fig. 1c–e). Other mutants were normally expressed in both EBV-B cells and HEK293 T cells.

### Consequence of *SOCS1* mutations on STAT pathway activation

**Fig. 2:**
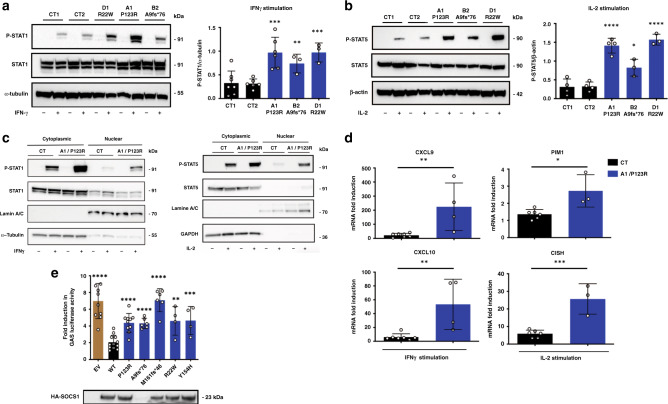
*SOCS1* mutations result in uncontrolled STAT pathways activation. **a**, **b** Left: Western blots (WB) of patients (A1, B2, and D1) and healthy controls (CT) derived EBV-B cells stimulated with IFN-γ (10^3^IU/ml for 1 h) (**a**) or IL-2 (10^4^IU/ml for 2 h) (**b**). Lysates were incubated with an antibody against tyrosine-phosphorylated STAT (P-STAT) or against total STAT, as indicated. Right: densitometric quantification of the phospho-STAT/α-tubulin or β-actin ratio upon stimulation. **a, b** Data are representative of *n* = 3 (patients B2 and D1), *n* = 4 (A1, IL-2 stimulation), and *n* = 6 (A1, IFNγ stimulation) independent experiments. Statistics (versus CT2): IFNγ stimulation, A1 *p* = 0.0008, B1 *p* = 0.0029, D1 *p* = 0.0002; IL-2 stimulation, A1 *p* < 0.0001, B1 *p* = 0.0118, D1 *p* < 0.0001. **c** The nuclear and cytoplasmic fractions of EBV-B cells from a control (CT) and from patient A1 after stimulation with IFN-γ for 1 h (left) or with IL-2 for 2 h (right) were tested by WB for the presence of P-STAT1 and P-STAT5, respectively. Anti-lamin A/C and anti-α-tubulin antibodies were used to normalize the amount of nuclear and cytoplasmic proteins. Data are representative of two independent experiments. **d** Real-time quantitative RT-PCR assays of *CXCL9* and *CXCL10* expression 6 h after stimulation with IFN-γ (left), and assays of *CISH* and *PIM1* expression 6 h after stimulation with IL-2 (right) in EBV-B cells from a CT and from patient A1. Results represent the fold-increased expression between stimulated and unstimulated states and are normalized to endogeneous GAPDH. Data are representative of *n* = 3 (IL-2 stimulation) and *n* = 4 (IFNγ stimulation) independent experiments performed in triplicate. Statistics: IFNγ stimulation, *CXCL9* *p* = 0.0094, CXCL10 *p* = 0.0063; IL-2 stimulation, *PIM1* *p* = 0.0105, *CISH* p = 0.0008. **e** Firefly luciferase activity in HEK293T cells transiently transfected with a gamma-activated sequence-driven IFN-γ reporter plasmid (GAS) and expression plasmids for WT or mutant SOCS1 proteins, and then stimulated with IFN-γ for 24 h. The results correspond to the fold-difference between the stimulated state and the unstimulated state. Results represent at least *n* = 4 independent experiments. All constructs were compared with WT SOCS1. Protein expression from the transfected plasmids was confirmed by immunoblotting the cell lysates (below, one representative result). Statistics: EV *p* < 0.0001, P123R *p* < 0.0001, A9FS*76 *p* < 0.0001, M161FS*46 *p* < 0.0001, R22W *p* = 0.0011, Y154H *p* = 0.0009. **a**, **b**, **d**, **e** Two-tailed *p* values were determined in an unpaired *t* est. Data indicate mean with SD. *P ≤ 0.05; **P ≤ 0.01; ***P ≤ 0.001; ****P ≤ 0.0001.

**Fig. 3:**
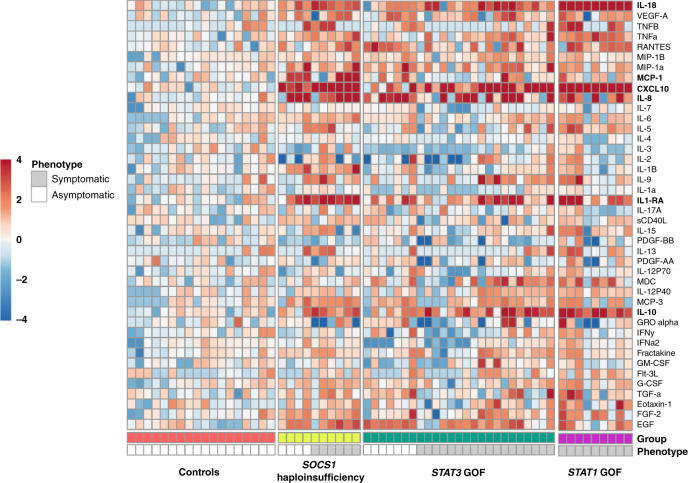
Inflammatory cytokine signature in serum from patients with *SOCS1* mutations. Cytokine array analysis of serum from patients with *SOCS1* insufficiency, controls, patients with *STAT1* GOF mutations, and patients with *STAT3* GOF mutations. Gray: symptomatic patients; white: asymptomatic carriers. Decimal logarithms of the values determined in a multiplex bead assay were color-coded as follows: for each individual cytokine, median values obtained in the 17 HCs were defined as 0 (white). The X-fold standard deviation above this median (0 to +4, coded in red) or below this median (0 to −4, coded in blue) is shown with the individual squares. The color code was arbitrarily truncated at ±4 SDs.

To examine the impact of the *SOCS1* mutations on the regulation of the STAT pathways in patients’ lymphocytes, EBV-B cells from A1, B2, and D1 were stimulated with various cytokines and STAT phosphorylation was determined as a surrogate of activation^3^. Of note, constitutive phosphorylation of the STAT1, STAT5 and STAT6 proteins was not detected. However, levels of IFN-γ-induced STAT1 phosphorylation, IL-2-induced STAT5 phosphorylation, and IL-4-induced STAT6 phosphorylation were higher in patients’ cells than in control cells (Fig. 2a, b and Supplementary Fig. 6). Consistently, we observed elevated nuclear translocation of P-STAT1 and P-STAT5 upon stimulation with IFN-γ and IL-2, respectively (Fig. 2c). Furthermore, expression of the IFN-γ inducible *CXCL9* and *CXCL10* genes and the IL-2-inducible *CISH* and *PIM1* genes was upregulated in patient cells following cytokine stimulation (Fig. 2d). Accordingly, no baseline hyperactivity was detected in patients’ primary monocytes but IFN-γ-induced STAT1 phosphorylation was also moderately but significantly higher than in control monocytes (Supplementary Fig. 7). Of note, the response to IL-21 was similar to that seen in controls in the cell line tested (A1, Supplementary Fig. 8). We next assessed the ability of mutant *SOCS1* variants expressed in HEK293T cells to suppress the transcription of IFN-γ inducible genes in a luciferase-based reporter assay by using a promoter containing an interferon-gamma-activated sequence (GAS). The overexpressed mutant proteins failed to suppress IFN-γ-mediated luciferase expression, whereas overexpression of the wild-type SOCS1 protein resulted in efficient inhibition (Fig. 2e). Addition of mutated proteins form of SOCS1 did not alter the inhibitory capacity of wild-type SOCS1 as observed with the four tested mutants (Supplementary Fig. 9), suggesting that the mutant proteins do not exert a dominant-negative effect. Taken together, these data indicate that SOCS1 haploinsufficiency confers hypersensitivity to, at least, IFN-γ, IL-2, and IL-4 cytokines. Consistently, an analysis of serum samples from patients revealed elevated levels of inflammatory mediators, such as IL-8, IL-10, IL-18, IL1-RA, CXCL10, and MCP-1 (Fig. 3). In contrast, the concentration of IFN-γ, IL-2, and IL-4 was not significantly increased. Interestingly, this cytokine signature was similar to that observed in individuals carrying gain-of-function (GOF) mutations in *STAT1* or *STAT3*.

### Effect of SOCS1 haploinsufficiency on T cells

**Fig. 4:**
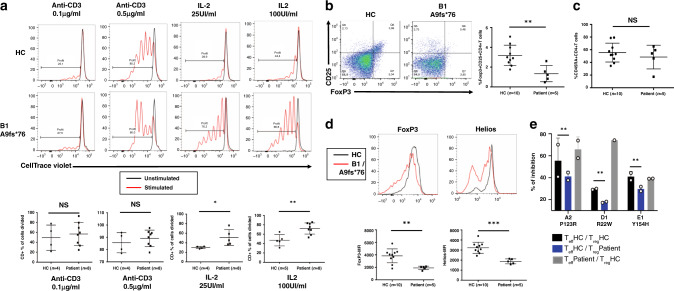
Impact of *SOCS1* mutations on T cell proliferation and on regulatory T cells. **a** Proliferation of T-cell blasts from healthy controls (HC) and patients. Day-10 T-cell blasts were stimulated or not with IL-2 (100 IU/ml), or anti-CD3-coated beads for 4 days. Proliferation was determined from the level of dilution of the CellTrace Violet dye. The panel shows proliferation of all T cells (CD3^+^). Top: representative histograms showing cell divisions of T-cell blasts (red peak) from an HC and from patient B1. Peak in black: unstimulated cells. The data are quoted as the percentage of cells having undergone at least one division. Bottom: the percentage of dividing cells from HCs and patients (data pooled from *n* = 4 independent experiments including a total of four HC, five patients with IL-2 25UI/ml and eight patients with anti-CD3 or IL-2 100UI/ml). Two-tailed *p* values were determined in an unpaired *t* test (IL-2 25UI/ml *p* = 0.0433, IL2 100UI/ml *p* = 0.0038, NS, not significant). **b** Left: a representative flow cytometry analysis of FoxP3 and CD25 markers in CD4+ T cells from an HC and from patient B1. Right: FoxP3^+^CD25^+^ CD4^+^ T cells (as a percentage of total CD4^+^ T cells) in the peripheral blood of HCs (*n* = 10) and patients (*n* = 5). **c** Naïve CD45RA^+^CD4^+^ T cells (as a percentage of total CD4^+^ T cells) in the peripheral blood of HCs (*n* = 10) and patients (*n* = 5). **d** Top: representative histograms of FoxP3 and Helios expression in CD4^+^CD25^+^CD127^low^ T cells from an HC (black) and patient B1 (red). Bottom: mean fluorescence intensity (MFI) of the respective T_reg_ cell markers in HCs (*n* = 10) and patients (*n* = 5). **b**–**d** Two-tailed *p* values were determined in a Mann–Whitney test (Panel B *p* = 0.0047; Panel D FoxP3 *p* = 0.008, Helios *p* = 0.0007, NS, not significant). **a**–**d** Data indicate mean with SD, and each dot corresponds to an individual. **e** Suppressive activity of regulatory T cells. VioBlue-labeled T_eff_ cells were cultured in the absence or presence of T_reg_ cells from HCs and patients (A2, D1, and E1). Proliferation was determined from the level of dilution of the VioBlue dye. Graphs indicate percentages of suppression from patients. Data are from three independent experiments in duplicate with a healthy control and indicated patient tested in pairs. Data indicate mean with SD of technical replicates due to the lack of cells for more experiments. Two-tailed *p* value was determined in a paired t-test between patients and controls (*p* = 0.0055). **a**–**e** *P ≤ 0.05; **P ≤ 0.01, ***P ≤ 0.001.

Given the lymphoproliferation observed in families B and C, we measured the proliferation of the patients’ T cells in response to various stimuli in vitro. The patients’ T cells proliferated more intensely than control T cells in response to stimulation with various concentrations of IL-2 but not in response to T-cell receptor stimulation with anti-CD3 antibodies (Fig. 4a). This hyperproliferation in response to IL-2 was not associated with increased expression of CD25, the alpha chain of the IL-2 receptor and a STAT5 target gene, neither on non-activated primary T cells nor on activated T cells blasts (Supplementary Fig. 10), suggesting an increased receptor internalization and/or shedding of CD25 in the presence of IL-2, or that SOCS1 deficiency may be counterbalanced by another mechanism yet to be determined.

In mice, Socs1 was previously shown to be involved in regulatory T cell (T_reg_) integrity and function, by maintaining Foxp3 expression and by suppressing Stat1 and Stat3 activation^9^. Moreover, *socs1* deletion in Foxp3^+^ cells in the mouse is sufficient to induce lymph node enlargement and splenomegaly^10^, prompting us to analyze T_regs_ in patients. The mean CD4^+^CD25^+^FOXP3^+^ T_regs_ frequency was lower in patients with SOCS1 haploinsufficiency (Fig. 4b). This reduction in T_regs_ frequency was not due to the expansion of memory CD4 T cells (Fig. 4c). With the exception of CTLA-4, the expression of several key T_reg_ markers (including FOXP3, HELIOS, and, to a lesser extent, CD25) was lower in patients’ T_regs_ (Fig. 4d and Supplementary Fig. 11). Furthermore, the T_regs_’ suppressive activity was moderately but significantly reduced in the patients’ T_reg_ cells (A2, D1, E1) as compared with control cells (Fig. 4e and Supplementary Fig. 12). Thus, SOCS1 deficiency leads to a defective T_reg_ compartment. Despite SOCS1’s putative role in CD4^+^ T cell differentiation^11^, we did not observe an obvious imbalance in Th1, Th2, Th17, and T follicular helper cells (Supplementary Fig. 13). No impact of SOCS1 deficiency on T cell apoptosis was observed (Supplementary Fig. 14).

### Immunological features in healthy mutation carriers

We identified five unaffected family members carrying the same *SOCS1* mutation as their affected relatives (Fig. 1a). At the time of the genetic analysis, these healthy carriers (B3, C2, D2, D3, E3) were 10, 54, 23, 52, and 62 years old, respectively. Four (C2, D2, D3, E3) were considered to be completely asymptomatic. One carrier (B3) developed severe asthma at 10 with an elevated serum IgE. Immunologic phenotyping performed in four carriers (B3, C2, D2, and E3) revealed similarities with the patients including low marginal zone and/or switch memory B-cell counts in three of them and elevated CD21^low^ CD38^low^B-cell count in two (Supplementary Table 1 and Supplementary Fig. 1). One (E3) of the two healthy carriers tested had autoantibodies (antinuclear and anti-SSA autoantibodies). Serum analysis from four of the carriers showed an inflammatory cytokine signature similar to patients (Fig. 3). Finally, IFN-γ-induced STAT1 phosphorylation in primary monocytes was higher than controls in two of the four tested healthy carriers (Supplementary Fig. 7). Taken together these data suggest that the immunological and functional phenotype is relatively penetrant even though the clinical phenotype is not.

### In vitro and ex vivo efficacy of JAK1/JAK2 inhibition

**Fig. 5:**
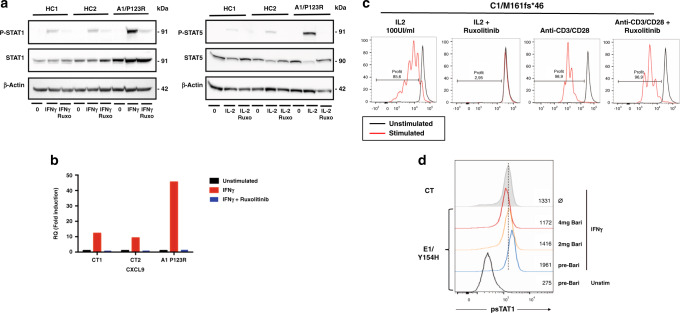
In vitro and ex vivo efficacy of JAK1/JAK2 inhibition. **a** EBV-B cells from HCs and from patient A1 were stimulated with IFN-γ (10^3^IU/ml for 1 h, left) and IL-2 (10^4^IU/ml for 2 h, right) in the presence or absence of ruxolitinib (Ruxo). Lysates were incubated with the indicated anti-P-STAT and anti-STAT antibodies, or anti-actin antibodies as a loading control. Data are representative of two independent experiments. **b** EBV-B cells from controls (CT) and from patient A1 were preincubated or not with ruxolitinib for 1 h, and stimulated for 6 h with IFN-γ. mRNA expression of the STAT1-regulated gene CXCL9 was determined in a quantitative RT-PCR assay. Results represent the fold-increased expression between stimulated and unstimulated states and are normalized to endogeneous GAPDH. Experiment performed once. **c** Effect of in vitro treatment with ruxolitinib on T-cell proliferation. T-cell blasts from patient C1 were stimulated with IL-2 (100 IU/ml) or anti-CD3/CD28 beads, in the presence or absence of ruxolitinib. The panel shows the proliferation of all T cells (CD3^+^). Data are representative of two independent experiments with cells from five patients (A1, A2, B1, B2, and C1). **d** Primary monocytes from healthy control (CT, filled line) and E1 before (blue line) and under treatment by 2 mg (orange line) or 4 mg (red line) of baricitinib were stimulated with IFN-γ for 15 min and STAT1 phosphorylation (P-STAT1) was determined by intracelular flow cytometry.

Given that *SOCS1* mutations are associated with uncontrolled JAK/STAT activation, we tested in vitro the effect of the JAK1/2 inhibitor ruxolitinib on IFN-γ-induced STAT1 phosphorylation and on the expression of the IFN-γ-regulated gene CXCL9. Both were found reduced in the presence of ruxolitinib (Fig. 5a, b). The same effect was observed on IL-2-induced STAT5 phosphorylation in EBV-B cells (Fig. 5a) and on IL-2-induced proliferation in T cells as well (Fig. 5c). In contrast, ruxolitinib had no effect on anti-CD3/CD28-induced T cell proliferation (Fig. 5c). Finally, one patient (E1) with systemic lupus erythematosus who was clinically well controlled under mycophenolate mofetil but because of disabling digestive intolerance was switched to baricitinib (JAK1/2 inhibitor) at 2 mg once daily followed by 2 mg twice daily (Table 1 and Supplementary Information). Following three months of treatment, the tolerance was good while a decrease in anti-DNA autoantibodies was observed (Supplementary Fig. 15). Furthermore, IFN-γ-induced STAT1 phosphorylation in patients’ primary monocytes was reduced under baricitinib as a function of the drug dosage given to the patient (Fig. 5d). These results indicate that SOCS1 haploinsufficiency is associated with the hyperactivation of JAK kinases through SOCS1-sensitive cytokine receptors—a finding thus with potential therapeutic value.

## Discussion

We found that SOCS1 haploinsufficiency is associated with dominantly inherited early onset autoimmunity and lymphoproliferation in ten patients from five unrelated families. These observations highlight a key role of SOCS1 in immune homeostasis in humans. We notably demonstrated that the *SOCS1* germline mutations leading to haploinsufficiency result in (i) higher JAK-STAT pathway activation in response to different cytokines, (ii) T cell hyperproliferation in response to IL-2, and (iii) a defective T_reg_ compartment. Our observation of multiple missense and frameshift mutations in patients with autoimmune features strongly suggests that these mutations are pathogenic. Furthermore, our present results in humans are in line with murine studies^4,9,12–14^. In particular, *Socs1*^−/−^ mice exhibited neonatal lethality due to excessive IFN-γ signaling and massive inflammatory infiltration^12^. Moreover, haploinsufficient mice survive into adulthood but develop a systemic autoimmune disease with age, reminiscent of human SLE^15^. Our findings are also in agreement with a recent study identifying, by whole-genome sequencing in a large primary immunodeficiencies cohort, three patients with autoimmune manifestations and SOCS1 haploinsufficiency^16^.

The assessment of the molecular consequences of single-gene mutations has provided a better understanding of the mechanisms underlying autoimmunity in humans^2^. Multiple defects can lead to a breakdown in immune tolerance, which includes the impairment of negative T-cell selection, defective lymphocyte apoptosis, a loss of B cell tolerance, abnormal T_reg_ function, uncontrolled activation of T cells, and accentuated immune effector cell functions^2^. Our observations in SOCS1 insufficiency provide mechanistic insight into the development of autoimmunity induced by cytokine hypersensitivity in immune cells following the loss of a downregulatory element. *STAT1* and *STAT3* GOF mutations could also be included in this category, given that they enhance cytokine signaling and function^17–19^. Interestingly, the clinical phenotypes of SOCS1 insufficiency and *STAT3* GOF mutations have some common features (including lymphadenopathy and autoimmune cytopenia) but also possibly differences (SLE in SOCS1 insufficiency vs. enteropathy and diabetes with *STAT3* GOF mutations)^20,21^. This may reflect the scope of the various cytokine signaling pathways involved^20,21^. We have not seen increased infection susceptibility in the *SOCS1* mutant patients, but this has to be confirmed in larger cohorts.

Several mechanisms can be proposed to explain autoimmunity in the context of SOCS1 insufficiency. In mice, deletion of *Socs1* in T cells is both necessary and sufficient to promote autoimmunity^13,22^. Mechanistically, this might be due to defective T_reg_ development and function in the absence of SOCS1, as previously shown in the mouse^9^ and here in humans. However, mice with a conditional knock-out of *Socs1* in T_regs_ exhibit a milder autoimmune phenotype than mice in which *Socs1* has been deleted in all T cells—suggesting that the cytokine hypersensitivity of conventional T cells also contributes to autoimmunity^9,10^. A role for SOCS1 in cytokine regulation of B cells is also plausible and may account, at least in part, for the abnormal B cell phenotype. Myeloid cells are plausibly also involved, since the autoimmune phenotype is more severe when *Socs1* is knocked out in both T cells and macrophages^23^. Moreover, SOCS1 deficiency in dendritic cells alone leads to the production of high levels of BAFF, the aberrant expansion of B cells, the production of autoreactive antibody, and systemic autoimmunity^13^. Hence, given the widespread expression of SOCS1 and the cytokines’ orchestrating role in the immune system, several cell types probably contribute to autoimmunity in the context of SOCS1 haploinsufficiency.

Of the 15 individuals with a heterozygous *SOCS1* mutation studied here, five (including four adults) were healthy carriers (33%). This incomplete clinical penetrance is frequently observed in autosomal-dominant primary immunodeficiencies caused by haploinsufficiency (e.g. *CTLA-4*^24,25^, *IKZF1/*IKAROS^26^ or in *TNFRSF6*/FAS deficiencies^27^, which have penetrance of 67%, 65%, and 30%, respectively). The age at onset differs in individuals carrying the same mutation, and the clinical manifestations sometimes appear as late as the sixth decade of life^25,26^. This incomplete penetrance may indicate the requirement for additional genetic^28^ or epigenetic events and/or the involvement of infectious/environmental events to induce full clinical expression of the disease^29^.

Several monogenic primary immunodeficiencies have been linked to an increased risk of malignancies^30^. This could be related to treatments with immunosuppressants and/or the gene product’s role in tumor surveillance^27^. Somatic loss-of-function mutations in *SOCS1* (including 2 mutated residues found in families A and E: P123 and Y154^7,8^) are frequently observed in B-cell or Hodgkin lymphoma, and are associated with activation of the JAK-STAT pathway in tumor cells^31–33^. It is noteworthy that one patient in family B developed Hodgkin lymphoma at the age of 34, whereas benign lymphoproliferation was seen in two other patients. This observation should prompt the physicians to carefully monitor patients with SOCS1 deficiency and their healthy carrier relatives.

SOCS1-insufficient patients have been treated with various nonspecific immunosuppressive agents, some of which are associated with toxic effects. Given the molecular etiology of the disease, treatment with drugs inhibiting the JAK signaling pathway might constitute a more specific treatment option^34^. We found indeed that in vitro, the JAK1/2 inhibitor ruxolitinib strongly inhibited T cell hyperproliferation and cytokine-mediated hyperphosphorylation of STAT1 and STAT5, without affecting the antigen-receptor-mediated response. These results were confirmed ex vivo in a patient with systemic lupus erythematosus treated by the JAK1/2 inhibitor baricitinib that was associated with an in vivo decrease of anti-DNA antibodies. Therefore, JAK inhibitors may be targeted therapies of value for SOCS1-insufficient patients. Moreover, SOCS1 itself is an attractive therapeutic target; SOCS1 agonists are being tested for efficacy in diseases mediated by IFN-γ, IL-2, or IL-4, including autoimmune and allergic disorders^35,36^.

In conclusion, our results show that SOCS1 haploinsufficiency is a recognized cause of early onset autoimmunity. Our results also provide mechanistic insights into the development of autoimmunity induced by cytokine hypersensitivity in immune cells—opening up avenues for personalized treatments in the field of autoimmunity.

## Methods

### Study participants

All study participants and their parents provided written informed consent. The study was performed in accordance with the 1975 Declaration of Helsinki and subsequent revisions, and was approved by the local institutional review board (CPP Ile de France II (Paris, France), the French Advisory Committee on Data Processing in Medical Research (Paris, France) and the Ethics committee, University of Freiburg, Germany (No. 409/16). The probands (patients A1, A2, B1, B2, C1, D1, and E1) were included in clinical studies of early onset autoimmune diseases by three independent institutions (Imagine Institute (Paris, France), Lyon Hospital (Lyon, France), and Freiburg Hospital (Freiburg, Germany)).

### Genetic analysis

Samples of DNA were prepared from the patients’ whole peripheral blood, using standard extraction methods. After quality control, genomic DNA (3 μg) intended for WES was captured using an in-solution enrichment method (Human All Exon v5—50 Mb, Agilent Technologies, CA, USA). Exome-enriched libraries (~20,000 targeted genes) were prepared on an automated system, using the NGSx robot (Perkin Elmer Inc, MA, USA) and the Bravo robot (Agilent Technologies, CA, USA) respectively, according to the manufacturers’ instructions (SureSelect, Agilent Technologies). After normalization and quality control, exome-enriched libraries were sequenced on a HiSEQ 2000 system (Illumina Inc., CA, USA) as paired-end 100b reads. The mean sequencing coverage was at least 60 to 70x for each sample. Image analysis and base calling were performed using the Illumina Real Time Analysis Pipeline. Sequence quality parameters were assessed daily throughout the 12-day sequencing run. Sequences were aligned with the hg19 reference human genome, using the Burrows-Wheeler Aligner. Downstream processing was carried out with the Genome Analysis Toolkit (GATK), SAMtools, and Picard, in line with documented best practices (http://www.broadinstitute.org/gatk/guide/topic?name=best-practices). Variants were called with the GATK Unified Genotyper. All calls with a read coverage of ≤2X or a Phredscaled SNP quality score of ≤20 were removed from the analysis. All variants were annotated using a software system developed by the Paris Descartes University Bioinformatics platform. All the annotation procedures were based on the latest release of the Ensembl database.

Whole-genome sequencing was performed by Edinburgh Genomics. Genomic DNA was prepared from whole blood samples from probands and parents, using the Qiagen Mini Kit according to the manufacturer’s instructions. Genomic DNA (gDNA) samples were evaluated for quantity using Quant-IT Picogreen reagent, Lambda Standard DNA, and a Spectramax XPS Gemini plate reader. Quality was evaluated using AATI Fragment analyzer and the Standard Sensitivity Genomic DNA Analysis kit. Samples with >1000 ng and a quality score of >5 passed the sample quality control. Sequencing libraries were prepared using a specific Illumina SeqLab TruSeq Nano High Throughput kit. A total of 200 ng of gDNA was normalized and sheared to a 450 bp mean insert size. TruSeq adapters were ligated onto the ends of each fragment prior to PCR amplification. Caliper GX Touch with an HT DNA HI SENSE Reagent Kit was used to produce mean fragment sizes of 530–950 bp. Libraries were normalized to 1.5 nM and sequenced at 300 pM. Demultiplexing was performed using bcl2fastq.

The raw sequencing data were quality-checked using FastQC and MultiQC, and subsequently aligned with the GRCh37 hs37d5 reference using the Edico Dragen V2 single-sample workflow. Indels and SNPs were called using the same workflow on an FPGA AWS instance. The resulting variant call format files were then uploaded to the Congenica platform via the latter’s Upload Client (v1.7.4) for annotation and causal variant prioritization. Variants were annotated using the Ensembl Variant Effect Predictor and scored for disease causality using Exomiser. For each variant in the proband, we determined the mode of inheritance in the family trio (de novo, autosomal recessive homozygous, autosomal recessive compound heterozygous, or X-linked recessive). DoNovoGear was used for the joint analysis of samples within a trio, in order to detect de novo mutations. Variants with qualifying genotypes under each mode of inheritance were ranked using the Exomiser combined score and evaluated for rarity (MAF in ExAC and UK10K), pathogenicity (Polyphen2 and SIFT scores), genic intolerance to functional variation (RVIS and pLI scores) and biological relevance in the context of the proband’s phenotype (using a Phenotype Consensus Analysis and a literature search). A rule-based scheme was used to identify candidate genes.

The pathogenicity of the identified variants was defined according to the minor allele frequency in the general population according to the GnomAD database (https://gnomad.broadinstitute.org/); and the Combined Annotation-Dependent Depletion score^37^.

Sanger sequencing was performed to confirm the next-generation sequencing results and to analyze each mutation’s familial segregation. The primers used for PCR are reported in Supplementary Table 1. Purified PCR products were directly sequenced using BigDye Terminators (version 1.1) and a 3500xL Genetic Analyzer (Applied Biosystems).

### Three-dimensional structure modeling

The model of the human SOCS1 3D structure was built on the basis of the experimental 3D structure of chicken SOCS1 (71 % sequence identity, pdb 6C7Y), according to the alignment given in Supplementary Fig. 2. The possible path of a phosphotyrosine peptide, represented in purple in Fig. 1d (bottom view), with the side chain of the phosphotyrosine shown, was taken from the experimental 3D structure of the SOCS3:gp130 complex (Protein Data Bank 2HMH). The model of the whole JAK1/SOCS1/elongin B/elongin C complex shown in Fig. 1d (top) is based on the chicken SOCS1/JAK1 (Protein Data Bank 6C7Y) and *X. laevis* SOCS1/Elongin B/Elongin C (Protein Data Bank 6C5X) 3D structures. Modeling was made using Modeller v9.22^38^ and 3D vizualisation using Chimera^39^.

### Cell culture

Peripheral blood mononuclear cells (PBMCs) were isolated using Ficoll separation (Lonza) and then cultured in Panserin 401 medium (Dutcher) with 5% human male AB serum (BioWest), 2 mM l-glutamine, 100 U/ml penicillin, and 100 μg/ml streptomycin (Thermo Fisher Scientific) at 37 °C in a humidified 5% CO_2_ incubator. Expansion of T cell blasts was obtained by incubating PBMCs for 72 h with CD3/CD28 beads (LifeTechnologies) in complete Panserin 401. After 3 days, dead cells were removed by Ficoll-Plaque density gradient, and blasts were expanded in complete Panserin supplemented with IL-2 (100 IU/ml). Epstein–Barr-virus (EBV)–transformed B lymphoblastoid cell lines (EBV-B cells) were generated from PBMCs obtained from patients (A1, B1, and D1) and HCs using standard methods and then cultured in RPMI 1640 (LifeTechnologies) supplemented with 10% fetal bovine serum (FBS) and penicillin/streptomycin. Human embryonic kidney (HEK) 293T cells were cultured in DMEM (LifeTechnologies) supplemented with 10% FBS and penicillin/streptomycin.

### Flow cytometry analysis

For surface staining, PBMCs (either freshly isolated or thawed after storage in liquid nitrogen) were washed with cold PBS and then incubated for 30 min at 4 °C (in the dark) with appropriate fluorochrome-labeled monoclonal antibodies. For intracellular staining, PBMCs were fixed and permeabilized after surface staining using the FoxP3 staining kit (eBioscience) according to the manufacturer’s instructions, and then incubated with anti-FOXP3 and anti-CTLA4 for 30 min on ice. The antibodies used for flow cytometry are listed in Supplementary Table 2. All samples were measured using flow cytometry (Becton Dickinson LSRFortessa X-20) and the data were analyzed using the FlowJo software (TreeStar).

### Expression plasmids, mutagenesis, and transfection experiments

Complementary DNA from WT human SOCS1 (*SOCS1*; NM_003745) was subcloned into the mammalian expression vector pCMV-HA-tagged (Clontech). The indicated *SOCS1* mutants were generated using the Q5 site-directed mutagenesis kit protocol (NEB). All cDNA sequences were confirmed by Sanger sequencing. Wild-type or mutant *SOCS1* plasmids were transfected into HEK293T cells by transient transfection using lipofectamine 2000 (Life Technologies).

### Western blot analysis

The EBV-B cell lines were exposed to various stimuli prior to protein extraction. The following optimal stimulation conditions were used: 60 min with 10^3^ IU/ml IFN-γ, 120 min with 10^4^ IU/ml IL-2, 60 min with 10 ng/ml IL-4, 45 min with 10 and 100 ng/ml IL-21, and 60 min with 100 ng/ml IL-6. For blocking experiments, cells were preincubated with ruxolitinib (500 nM) for an hour prior to stimulation. Cell activation was blocked with cold 1X PBS, and then lysed in RIPA lysis buffer supplemented with a protease and phosphatase inhibitor cocktail (ThermoFisher Scientific). The extracted proteins were quantified using a Pierce BCA assay kit (ThermoFisher Scientific), subjected to SDS-PAGE, and transferred to a PVDF membrane. For the extraction of nuclear and cytoplasmic proteins, EBV-B cells were stimulated with INFγ for 1 h or IL-2 for 2 h and extracted with NE-PER Nuclear and Cytoplasmic Extraction Regents (ThermoFisher Scientific), according to the manufacturer’s instructions. After blocking, the membrane was incubated overnight with the following primary antibodies: anti-SOCS1 (Cell Signaling Technology), anti-phosphorylated STAT1^Y701^ (P-STAT1, Cell Signaling Technology), anti-STAT1 (Cell Signaling Technology), anti-P-STAT3^Y705^ (Cell Signaling Technology), anti-STAT3 (Cell Signaling Technology), anti-P-STAT5^Y694^ (Cell Signaling Technology), anti-STAT5 (Cell Signaling Technology), anti-P-STAT6^Y641^ (Cell Signaling Technology), anti-STAT6 (Cell Signaling Technology), anti-α-tubulin (Cell Signaling Technology), anti-β-actin (ThermoFisher Scientific), anti-GAPDH (Cell Signaling Technology), anti-HA (Cell Signaling Technology), and anti-lamin A/C (Cell Signaling Technology). The results were visualized by chemiluminescence using species-specific HRP-linked secondary antibodies.

### Real-time quantitative RT-PCR

The EBV-B cells were stimulated for 6 h with 10^3^ IU/ml IFN-γ or 10^4^ IU/ml IL-2. Total RNA was isolated using the RNeasy Mini Kit (Qiagen), and cDNA was prepared using the Quantitect Reverse Transcription Kit (Qiagen) after depletion of genomic DNA. Real-time quantitative PCR was performed using the LightCycler VIIA7 System (Roche). Gene transcript levels were normalized against an endogenous *GAPDH* control. The results were quoted as the fold-increase in expression, relative to the unstimulated state. Each experiment was performed in triplicate in at least two independent experiments.

### T-helper cell cytokine assays

Peripheral blood mononuclear cells from patients and HCs were stimulated ex vivo with phorbol myristate acetate (50 ng/ml; Sigma-Aldrich) and ionomycin (1 µg/ml; Sigma-Aldrich) for 4 h in the presence of brefeldin A (GolgiPlug, 1:1000; BD Biosciences), surface-stained, fixed/permeabilized (with a Cytofix/Cytoperm plus Kit, BD Biosciences) and stained with anti-IL-4, IL-17A and IFN-γ antibodies (Supplementary Table 2). The fraction of Th2, Th17, and Th1 cells was respectively determined (using flow cytometry) as the percentages of IL-4^+^, IL17A^+^, and IFN-γ^+^ cells among CD4^+^ CD45RO^+^ cells.

### STAT 1 phosphorylation in monocytes

PBMCs of patients and healthy controls were left unstimulated or stimulated with IFN-γ (1000 IU/ml) for 15 min and subsequently fixed, permeabilized (Phosflow Lyse/Fix buffer, BD and Phosflow Perm buffer III, BD) and stained with anti-CD14 or anti-CD33 and anti-P-STAT1^Y701^ antibodies (Supplementary Table 2). The extent of P-STAT1 upon stimulation was determined by flow cytometry as the mean fluorescence intensity (MFI) in CD14^+^ cells or CD33^+^ cells.

### Luciferase reporter assays

HEK293T cells were dispensed into a 96-well cell-culture plate and transiently cotransfected using Lipofectamine 2000 (Life Technologies) with a luciferase reporter vector under the control of the interferon-gamma-activated (GAS) promoter (Promega), a *Renilla* control vector (Promega), and plasmids expressing either WT *SOCS1*, the various mutant *SOCS1* cDNAs, or a mock vector. 6 h after transfection, the cells were transferred back into medium containing 10% FBS and cultured for 24 h. Transfected cells were then stimulated (or not) with IFN-γ (10^3^ IU/ml) for 24 h and subjected to luciferase assays with the Dual-Glo luciferase assay system (Promega). Experiments were performed in triplicate, and firefly luciferase activity was normalized against the level of *Renilla* luciferase activity. The data are expressed as fold-induction, relative to unstimulated cells.

### Proliferation assay

Expansion of T cell blasts was obtained by incubating PBMCs for 72 h with anti CD3/CD28 beads in complete Panserin 401 medium. After 3 days, dead cells were removed by Ficoll-Plaque density gradient, and T-cell blasts were expanded in complete Panserin supplemented with IL-2 (100 IU/ml). Day 10 T-cell blasts were starved of IL-2 for 72 h, washed, incubated with CellTrace violet reagent (Invitrogen) for 8 min at 37 °C in the dark, and washed twice more. A total of 2 ×10^5^ cells were seeded into 96-well plates and subjected to different stimuli (plate-bound anti-CD3 antibody (Invitrogen), soluble anti-CD3/CD28 beads (eBioscience), IL-2 at the concentration indicated in the figures) in the presence of absence of ruxolitinib (500 nM). The cells were cultured for 4 days, washed with PBS, and stained with anti-CD3, CD4, CD8, CD25, and CD69 antibodies prior to flow cytometry measurement.

### T_reg_ cell suppression assay

PBMCs were incubated for 30 min at 4 °C with specific, labeled monoclonal antibodies, washed, and then sorted using an ARIA II cytometer (BD Biosciences). Naïve T effector cells (Teff) were defined as CD3^+^CD4^+^CD25^−^CD127^+^CD45RA^+^ T cells, Tregs were defined as CD3^+^CD4^+^CD25^+^CD127^−^ T cells, and dendritic cells (DCs) were defined as CD3-CD4-CD14-CD16-CD11c^+^ cells. After cell sorting, Teff was washed and stained with CellTrace CFSE. Next, the cells were washed, incubated with DCs (Teff:DC ratio 1:0.4), Tregs (Teff:Treg ratio 1:0.5) and stimulated by Staphylococcus enterotoxin E (0.2 ng/ml). The cells were cultured for 5 days in complete Panserin medium (Dutscher) in 96-well plates and the proportion of proliferating cells was measured with a MACSquant system (Miltenyi). The data were analyzed with FlowJo software. The percentage of suppression was calculated with the following formula: [log2(y) of (Teff cells alone) – log2(y) of (Teff + Treg cells])/log2(y) of (Teff cells alone) × 100. The *y* value corresponds to the mean fluorescence intensity of the CFSE of the whole Teff cell population, divided by the mean fluorescence intensity of the CFSE of undivided Teff cells^40^.

### Cytokine assays

Serum levels of the cytokines EGF, eotaxin-1, FGF-2, Flt-3L, fractalkine, G-CSF, GM-CSF, GRO(alpha), IFN-alpha2, IFN-γ, IL-1α, IL-1β, IL-1ra, IL-2, IL-3, IL-4, IL-5, IL-6, IL-7, IL-8, IL-9, IL-10, IL-12 (p40), IL-12 (p70), IL-13, IL-15, IL-17A, IL-18, IP-10, MCP-1, MCP-3, MDC, MIP-1α, MIP-1β, PDGF-AA, PDGF-AB/BB, RANTES, sCD40L, TGFα, TNFα, TNFβ, VEGF-A were determined using a multiplex bead assay (Human Cytokine Array/Chemokine Array 42-Plex with IL-18 (HD42), Eve Technologies; Canada).

### Statistics

All statistical analyses were performed using GraphPad Prism (version 6). As indicated in the Figures, results were analyzed in a two-tailed, unpaired or paired Student’s *t* test, or a two-sided Mann–Whitney U test. For all analyses, the threshold for statistical significance was set to *p* < 0.05.

### Reporting summary

Further information on research design is available in the Nature Research Reporting Summary linked to this article.

## Supplementary information

Supplementary Information

Reporting Summary

## Data Availability

SOCS1 variants sequences are deposited in the ClinVar database [https://www.ncbi.nlm.nih.gov/clinvar?term=603597[MIM]] with the following accession codes SCV001430913, SCV001430914, SCV001430915, SCV001430916, SCV001430917. Gnomad database (https://gnomad.broadinstitute.org/) was used to calculate the minor allele frequency of all the mutations. PopViz webserver (http://shiva.rockefeller.edu/PopViz/) was used to calculate the CADD score and the mutation significance (MSC) cutoff of all the mutations. The datasets generated and/or analyzed during the current study are available from the corresponding authors on reasonable request.  Source data are provided with this paper.

## Source data

Source Data
